# *SPOP* mutation drives prostate neoplasia without stabilizing oncogenic transcription factor ERG

**DOI:** 10.1172/JCI96551

**Published:** 2017-12-04

**Authors:** Jonathan Shoag, Deli Liu, Mirjam Blattner, Andrea Sboner, Kyung Park, Lesa Deonarine, Brian D. Robinson, Juan Miguel Mosquera, Yu Chen, Mark A. Rubin, Christopher E. Barbieri

**Affiliations:** 1 Department of Urology, New York Presbyterian Hospital,; 2 HRH Prince Alwaleed Bin Talal Bin Abdulaziz Alsaud Institute for Computational Biomedicine,; 3 Sandra and Edward Meyer Cancer Center, and; 4 Department of Pathology and Laboratory Medicine, Weill Cornell Medicine, New York, New York, USA.; 5 The Caryl and Israel Englander Institute for Precision Medicine of Weill Cornell Medicine, and New York-Presbyterian Hospital, New York, New York, USA.; 6 Human Oncology and Pathogenesis Program, and; 7 Department of Medicine, Memorial Sloan Kettering Cancer Center, New York, New York, USA.; 8 Department of Medicine, Division of Hematology and Medical Oncology, Weill Cornell Medicine, New York, New York, USA.; 9 Department of BioMedical Research, University of Bern, Bern, Switzerland.

**Keywords:** Oncology, Oncogenes

## Abstract

Nearly 50% of prostate cancers harbor gene fusions that lead to overexpression of the transcription factor ERG, while a mutually exclusive 10% of prostate cancers harbor recurrent mutations in the gene encoding the E3 ubiquitin ligase SPOP. Recent reports suggest that SPOP acts as a ubiquitin ligase for ERG and propose that ERG stabilization is the oncogenic effector of SPOP mutation. Here, we used human prostate cancer samples and showed that the vast majority of human SPOP-mutant cancers do not express ERG. Comparison of SPOP-mutant and ERG-fusion organoid models showed evidence of divergent, rather than common, transcriptional programs. Furthermore, expression of prostate cancer–associated *SPOP* mutations in genetically engineered mouse models of SPOP-mutant prostate cancer did not result in the expression of ERG protein in histologically normal prostate glands, high-grade prostatic intraepithelial neoplasia, invasive adenocarcinoma, or prostate organoids. In summary, we found no evidence that ERG is an effector of *SPOP* mutation in human prostate cancer or mouse models.

## Introduction

Over the past decade, there has been remarkable progress in the molecular classification of prostate cancer, with the delineation of multiple distinct subclasses defined by recurrent genomic aberrations. Recurrent gene fusions in the oncogenic transcription factor *ERG*, present in 40% to 50% of prostate cancers, define one such subclass, while a second distinct class, comprising 10% of prostate cancers, is defined by recurrent mutations in *SPOP* (1, 2). *SPOP* mutations and *ERG* rearrangements show near complete mutual exclusivity across multiple independent cohorts representing thousands of prostate cancer samples (1, 3–5).

*SPOP* encodes the substrate recognition component of a CUL3-based E3 ubiquitin ligase. Recent reports demonstrated that the SPOP-CUL3 complex can act as a ubiquitin ligase for ERG (6, 7). These studies present compelling biochemical evidence, using in vitro models, that SPOP can interact with ERG, facilitate its ubiquitination, and promote degradation in a degron-specific manner. One of the conclusions of these studies is that mutual exclusivity between *SPOP* mutation and *ERG* rearrangement is due to functional redundancy, and that ERG stabilization is a critical downstream mediator of the oncogenic effects of *SPOP* mutation in prostate cancer (6–8).

Here, we test this hypothesis using human prostate cancer specimens and genetically engineered mouse models of *SPOP*-mutant prostate cancer. Our data demonstrate that in both human cancer specimens and mouse models where SPOP mutation drives prostate neoplasia, ERG is not expressed, and we see no evidence of activation of ERG target genes. Taken together, our findings argue against *SPOP* mutation and *ERG* rearrangement as functionally redundant events.

## Results and Discussion

### Murine SPOP-driven prostate cancer does not express ERG.

We recently reported that mice expressing SPOP-F133V in the prostate display a high prevalence of high-grade prostatic intraepithelial neoplasia (HG-PIN) with striking nuclear atypia in combination with conditional heterozygous *Pten* loss (*Pten^L/+^*), which has a minimal phenotype by itself (9). SPOP-F133V in combination with homozygous *Pten* deletion (*Pten^L/L^*) (which on its own results in diffuse HG-PIN), develop highly prevalent invasive prostate adenocarcinoma (9).

**Figure 1:**
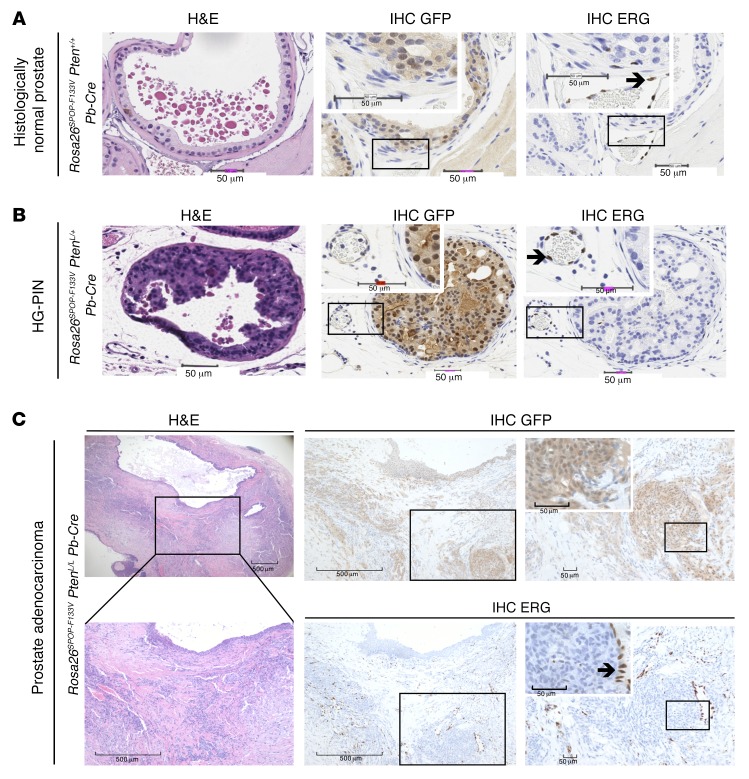
SPOP mutation does not result in ERG protein expression by immunohistochemistry in normal or neoplastic murine prostate. (**A**) Histologically normal prostate from mice conditionally expressing SPOP-F133V in the prostate (*Rosa26^SPOP-F133V^* *Pten^+/+^ Pb-Cre*). **A** and **B** scale bars: 50 μm. (**B**) SPOP-mutation-driven murine HG-PIN (*Rosa26^SPOP-F133V^* *Pten^L/+^ Pb-Cre*). (**C**) SPOP-mutation-driven murine prostate adenocarcinoma. (*Rosa26^SPOP-F133V^* *Pten^L/L^ Pb-Cre*). Insets show ERG staining in endothelial cells (arrow) adjacent to SPOP-mutant-expressing prostate cells. SPOP-F133V transgenic expression confirmed by GFP expression. A minimum of 3 mice were utilized for each condition. Representative sections are shown. Scale bars: 50 μm in right images of **C**, 500 μm in left and center images of **C**.

To determine the role of ERG in phenotypes observed in SPOP-mutant mouse models, we examined ERG protein expression by immunohistochemistry (IHC) using a well-characterized antibody (10). We did not observe ERG expression in histologically normal prostate epithelial cells (*Rosa26^SPOP-F133V^* *Pten^+/+^ Pb-Cre*) where SPOP-F133V, marked by GFP expression, is expressed (Figure 1A). As expected, ERG was readily detectable in endothelial cells (Figure 1A). In *Rosa26^SPOP-F133V^* *Pten^L/+^* *Pb-Cre* mouse prostates with HG-PIN driven by SPOP mutation (9), we again saw no evidence of ERG protein expression by IHC (Figure 1B). Similarly, in *Rosa26^SPOP-F133V^* *Pten^L/L^* *Pb-Cre* mouse prostates, in which SPOP mutation drives prostatic adenocarcinoma, ERG was not detectable by IHC in prostate cells expressing mutant SPOP (Figure 1C).

**Figure 2:**
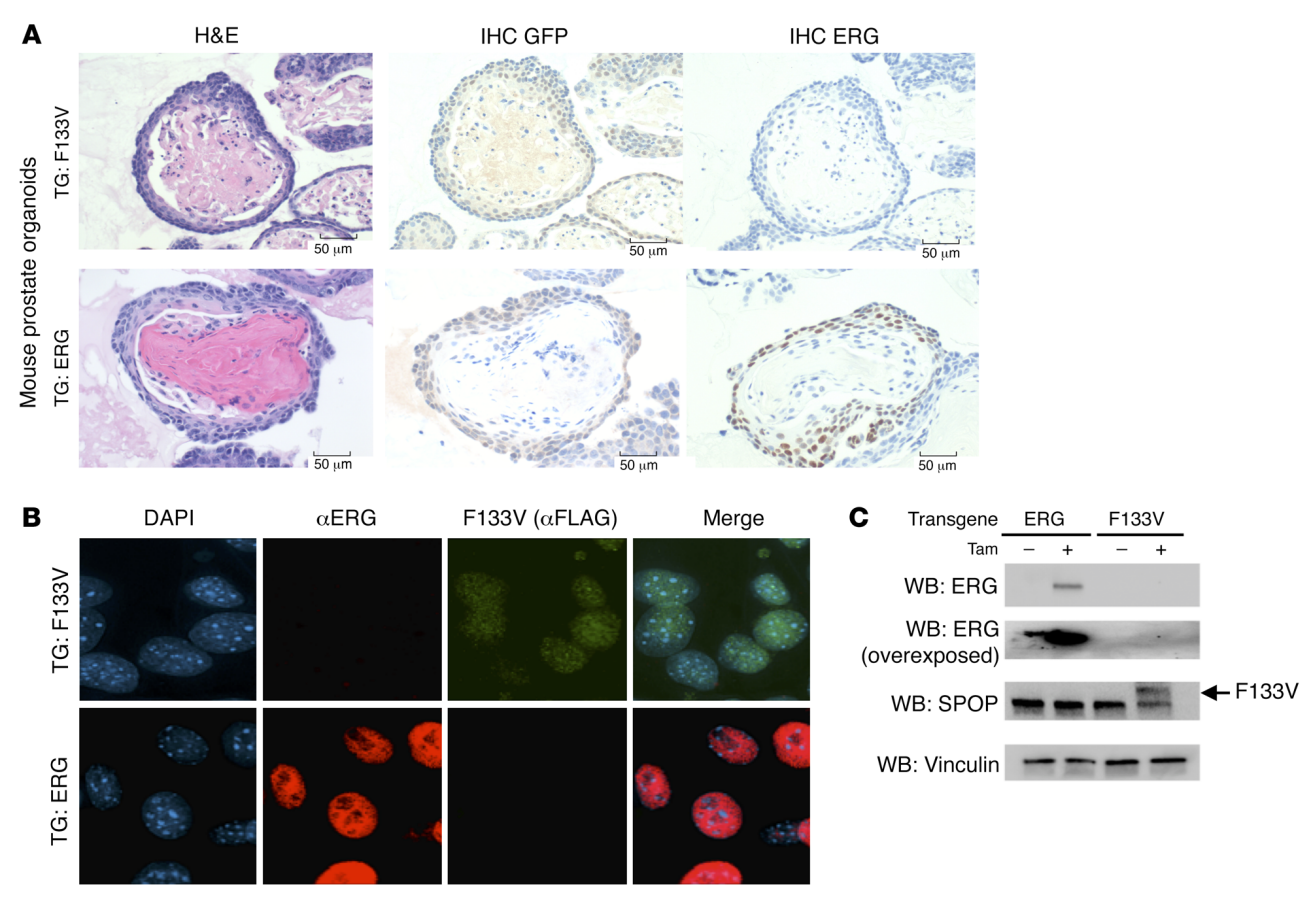
Murine prostate organoids expressing SPOP-F133V show no evidence of ERG upregulation. (**A**) ERG IHC in mouse prostate organoids expressing mutant SPOP (top) or ERG fusion as a positive control (bottom). SPOP-F133V transgenic expression confirmed by GFP expression. Scale bars: 50 μm. (**B**) ERG immunofluorescence in mouse prostate cells expressing SPOP-F133V (top) or ERG as a positive control (bottom). Original magnification: ×1000. (**C**) ERG protein expression by Western blot in organoids expressing SPOP-F133V or ERG as a positive control. Representative image of 3 experiments shown. Tam, tamoxifen; TG, transgene; WB, Western blot.

We next determined if SPOP mutation increased ERG expression in prostate organoids from *Rosa26^SPOP-F133V^* *Pten^L/+^ T2-Cre* mice. When mutant SPOP was expressed following induction with tamoxifen, we saw no evidence of ERG expression when assessed by IHC, immunofluorescence, or Western blot (Figure 2, A–C).

### ERG overexpression does not drive human SPOP-mutant prostate cancer.

**Figure 3:**
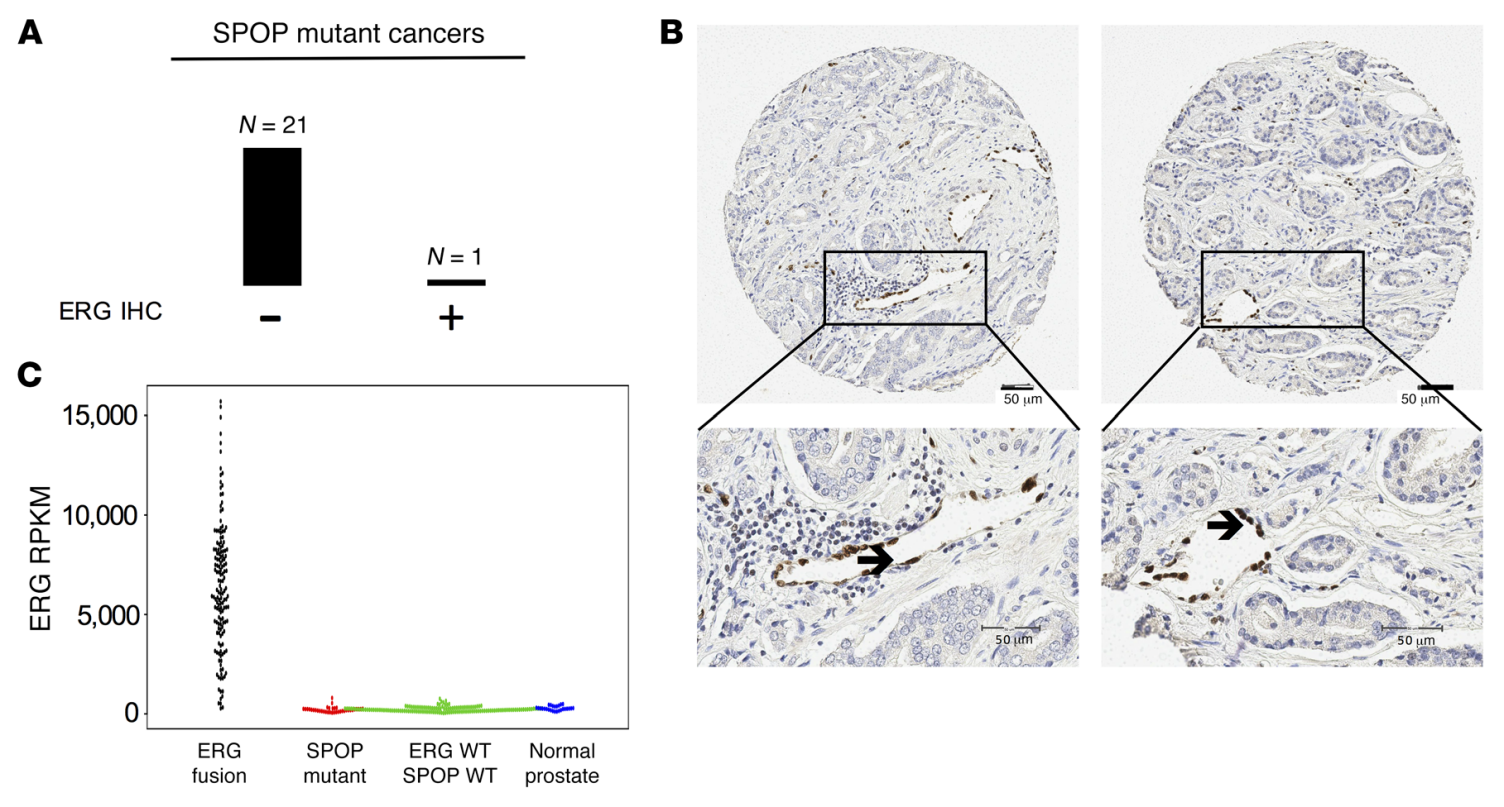
SPOP-mutant human prostate cancers do not express ERG. (**A**) Results of ERG IHC in 22 human prostate cancers where SPOP mutation was detected. (**B**) Images of 2 SPOP-mutant cancers not expressing ERG. Arrow denotes ERG-expressing endothelial cells. (**C**) Beeswarm plots of *ERG* transcript level in reads per kilobase of transcript per million mapped reads (RPKM) across prostate cancer molecular subclasses: 175 ERG-fusion samples, 37 SPOP-mutant samples, 121 ERG/SPOP wild-type samples from 333 TCGA human prostate cancer samples, and 23 TCGA normal samples. Scale bars: 50 μm.

A critical component of the studies defining ERG as deregulated by SPOP mutation was the demonstration of human prostate cancers harboring both SPOP mutation and ERG protein overexpression. We identified 22 SPOP-mutant prostate cancer samples and examined ERG expression by IHC. Only one of these cancers expressed detectable ERG by IHC (Figure 3, A and B, and Supplemental Figure 1, A and B; supplemental material available online with this article; https://doi.org/10.1172/JCI96551DS1), which was heterogeneous (2 out of 3 cores ERG positive).

Another central tenet of the hypothesis that SPOP stabilizes ERG in human prostate cancer is that baseline levels of ERG protein are present that accumulate in the presence of mutant SPOP. However, multiple studies have suggested that in the absence of gene fusion, ERG is not expressed in benign prostate cells (10–13). As seen in Figure 3C, *ERG* mRNA is expressed below levels that are generally considered adequate for expression in SPOP-mutant cancer.

**Figure 4:**
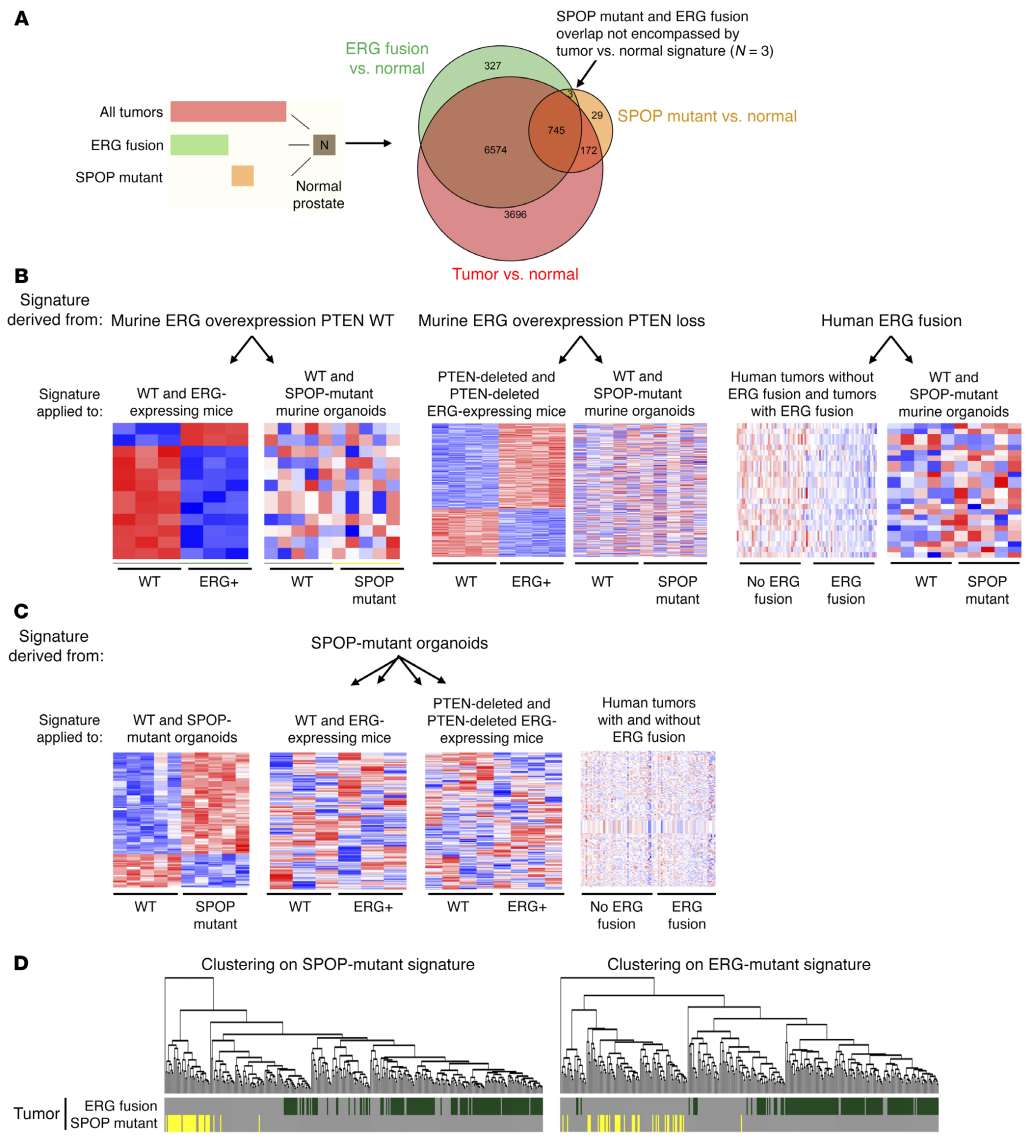
SPOP-mutant and ERG-fusion human prostate cancer share minimal common features. (**A**) Overlap of expression signatures from ERG-fusion tumors, SPOP-mutant tumors, and all tumors, as compared with normal prostate. (**B**) Heatmaps of *ERG* gene expression signatures in mouse and human prostate tissue with and without *ERG*-fusion expression, and SPOP-mutant and SPOP wild-type prostate organoids. (**C**) Heatmaps of SPOP gene expression signature in SPOP-mutant and SPOP wild-type organoids, and ERG-expressing and wild-type mouse prostate tissue from PTEN wild-type and PTEN-deleted mice, and ERG-fusion and ERG-fusion-negative human prostate cancer samples. (**D**) Unsupervised clustering of TCGA human prostate cancer samples based on the SPOP-mutant (left) and ERG-fusion expression signatures (right).

The studies by Gan et al. and An et al. have also suggested that ERG- and SPOP-mutant cancers share similar gene expression signatures (6, 7). We examined the overlap of signatures from The Cancer Genome Atlas (TCGA) by analyzing whether expression similarities between SPOP-mutant and ERG-rearranged tumors were unique to these tumor types, or rather represented a tumor versus normal signature. As seen in Figure 4A, virtually all of the overlap in gene signatures was accounted for by this tumor versus normal signature. Only 3 genes overlapped between SPOP-mutant and ERG-fusion signatures that were not incorporated in the tumor versus normal signature.

We next sought to determine if SPOP-mutant organoids, which recapitulate the human SPOP-mutant signature (9), expressed ERG transcriptional signatures. We saw that SPOP-mutant organoids do not cluster according to ERG-mutant signatures (Figure 4B). Similarly, ERG-mutant mouse prostates and human tumors do not cluster according to SPOP-mutant signatures (Figure 4C). Indeed, when clustered within prostate cancers, SPOP-mutant cancers cluster closer to non–SPOP-, non–ERG-rearranged cancers than ERG-rearranged cancers (Figure 4D and Supplemental Table 1).

Mutually exclusive genomic events can represent several types of functional interactions, including synthetic lethality, biological divergence, or functional redundancy. Here, we present evidence against ERG stabilization downstream of SPOP mutation being an important carcinogenic mechanism in prostate cancer. Using in vivo and in vitro models, we show that expression of SPOP-F133V, the most commonly mutated residue in prostate cancer, results in no detectable ERG protein expression in prostate cells. This is the case even in contexts where SPOP-F133V drives clear oncogenic phenotypes (9). We also see no evidence of stabilization of transgenic ERG by SPOP-F133V in these contexts. These data strongly argue against stabilization of ERG as a critical downstream mediator of the effects of mutant SPOP in prostate cancer.

In human cancers, consistent with prior reports, we show that the vast majority of SPOP-mutant tumors show no evidence of ERG protein expression. While we, as well as the previous studies, did identify one tumor in which both SPOP mutations and ERG protein expression were detected, this tumor had a marginal level of ERG expression that was present only in 2 of 3 tumor cores. This heterogeneity is not surprising, as prostate cancer is generally a multifocal disease, with the vast majority of glands at radical prostatectomy containing more than one cancer focus (14, 15). Heterogeneity, and the innate challenges of sampling human prostate cancers, can confound analyses of cooccurrence of molecular events. As stated above, it is possible that 2 clonally distinct foci of prostate cancer, with distinct molecular features, can spatially comingle (16). These so-called collision tumors are relatively common in prostate cancer, and complicate interpretation of molecular characterization. We have previously shown detailed characterization (using microdissection) on one such cancer, where an SPOP-mutant cancer collided with an ERG-expressing tumor (3). This alternative hypothesis should be ruled out before concluding that mutually exclusive events, like SPOP mutation and ERG protein expression, are occurring in the same cells.

Clonality and in situ studies support both ERG rearrangement and SPOP mutation as early events in the natural history of prostate cancer (2, 3, 17). Whether these mutations affect downstream prostate cancer progression is unclear (2, 17). ERG-positive tumors have been reported to be associated with younger age and lower prostate serum antigen (PSA); however, the prognostic significance of ERG rearrangement has been variable across cohorts (17, 18). To date, SPOP-mutant tumors have not been clearly associated with any clinical features; however, it is possible that additional cohorts with increased sample sizes will provide further insight into differential features among these subclasses (4).

Our study reinforces prior work that has shown that SPOP-mutant and ERG-rearranged tumors have different patterns of point mutations (1, 3), somatic copy number aberrations (1, 4), genomic rearrangements (1, 19, 20), DNA methylation (1), and gene expression (1). Collectively, these data support the hypothesis that ERG rearrangement and SPOP mutation represent divergent events leading to distinct biological classes of prostate cancer.

## Methods

### Mice.

Gene targeting was performed as previously described (9). For generation of prostate-specific SPOP-F133V expression, *Rosa26^SPOP-F133V^* mice were crossed with previously described *Pten^L/+^ PbCre4* mice. Only male *PbCre4*-positive mice were used to carry the *PbCre4* allele. For organoid studies, we crossed mice expressing TMPRSSS2-CreERT2 with mice expressing SPOP-F133V or ERG on the Rosa26 locus (9, 19). All described mice are in the C57BL/6 background.

Prostate tissue was harvested from mice euthanized using CO_2_, and samples were fixed in 4% formalin overnight and embedded in paraffin. Tissue paraffin embedding, sectioning, and staining with hematoxylin and eosin (H&E) and IHC were performed by the translational research program at Weill Cornell Medicine (WCM) Pathology and Laboratory Medicine. ERG IHC was performed using the Abcam EPR3864 clone, which has previously been characterized in detail by our group and others (10, 12, 21). Endogenous controls (endothelial cells and lymphocytes) as well as known ERG-positive samples were used as positive controls. GFP IHC was performed with Abcam AB13970. Sections were reviewed by a board-certified genitourinary pathologist with specific expertise in mouse models of human prostate cancer (B.D. Robinson or K. Park).

### Mouse prostate organoid generation and experiments.

Prostate tissue was extracted from euthanized mice, digested, and organoids grown as previously described (9, 22). CreERT2 was activated by adding 1 μM 4-hydroxytamoxifen (Sigma-Aldrich, T176) to the medium overnight or using adenovirus expressing Cre. Either GFP or blind (for control cells) sorting was performed on a BD FACSAria II (BD Life Sciences).

### Statistics.

Gene expression signatures were derived from differentially expressed genes among ERG-fusion, SPOP-mutant, and all tumors, as compared with normal samples, by using the Wilcoxon signed-rank test after transforming the RSEM via log_2_(RSEM + 1) from TCGA human prostate cancer and normal samples. Multiple-hypothesis testing was considered by using Benjamini-Hochberg (FDR ≤ 0.001) correction. Unsupervised clustering of TCGA human prostate cancer samples were generated based on SPOP-mutant and ERG-fusion signatures. The SPOP-mutant signature was derived from the differentially expressed genes between SPOP-mutant and SPOP/ERG wild-type samples using the Wilcoxon signed-rank test after transforming the RSEM via log_2_(RSEM + 1) using an FDR of 0.0001 or lower. The ERG-fusion signature was derived from differentially expressed genes between ERG-fusion and SPOP/ERG wild-type samples following a method similar to that described above.

### Study approval.

Relevant human studies were approved by the WCM Institutional Review Board (protocol 1007011157R007). Informed consent was obtained from patients for use of pathologic tissue. Mouse studies were approved by the WCM Institutional Care and Use Committee under protocol 2015-0022.

## Author contributions

JS, DL, AS, JMM, and CEB designed the research studies. JS, LD, and MB conducted in vitro and in vivo experiments. KP and BDR performed pathology review. DL and AS performed computational analyses. MB performed sequencing. YC and MAR provided unique critical reagents and helped design experiments. All authors contributed to writing the manuscript.

## Supplementary Material

Supplemental data

Supplemental Table 1

## Version 1 12/04/2017

Electronic publication

## Version 2 01/02/2018

Print issue publication
