# Supplementary material for: SPOP mutation drives prostate neoplasia without stabilizing oncogenic transcription factor ERG
Source: J Clin Invest. 2017 Dec 4;128(1):381–6. doi: 10.1172/JCI96551 (PMC5749531; doi:10.1172/JCI96551)
Supplement: Supplemental data [file jci-128-96551-s001.pdf]

A.

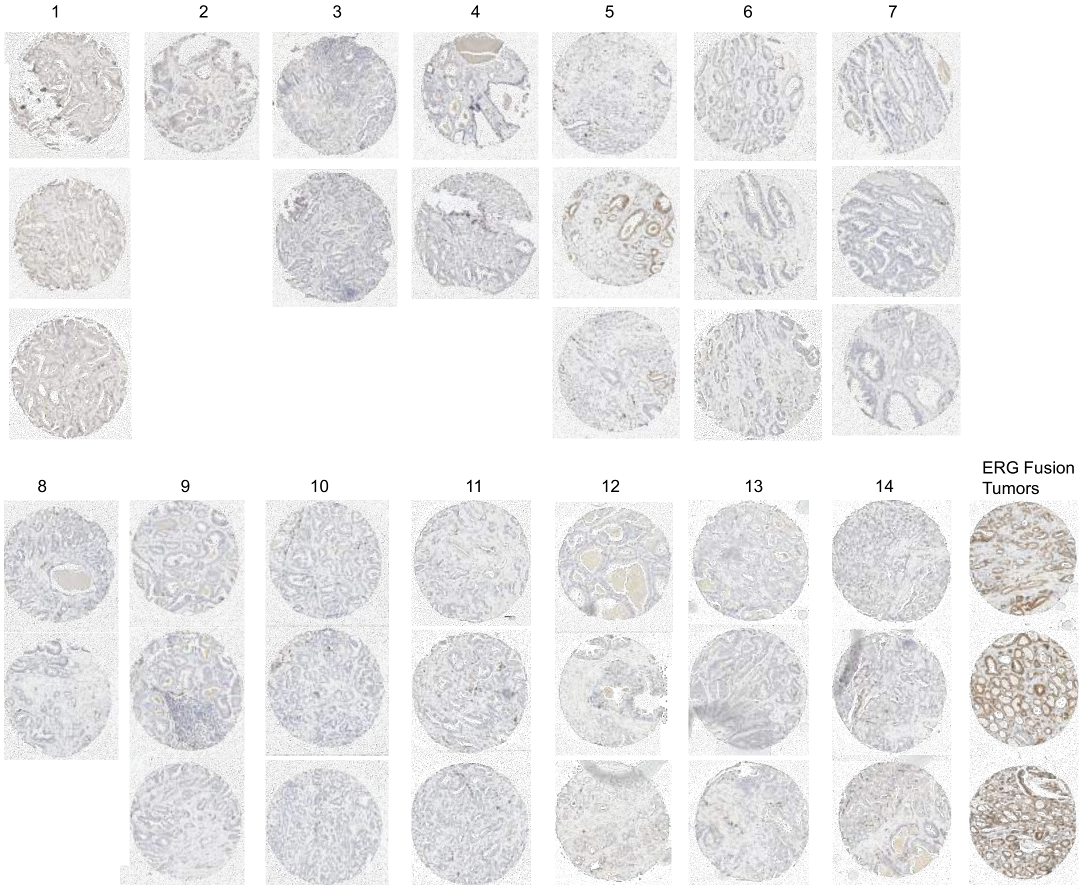

B.

SPOP mutation	Number of tumors
F133V	6
F133L	4
F133S	1
Y87C	2
Y87N	1
F102C	2
F102V	1
W131G	2
D130N	1
K129E	1
S119N	1

Supplemental Figure 1

A. ERG IHC in SPOP mutant human tumors. Up to 3 cores shown per tumor.
B. SPOP mutations detected in 22 SPOP mutant tumors subjected to ERG IHC.
